# Observations of enhanced internal waves in an area of strong mesoscale variability in the southwestern East Sea (Japan Sea)

**DOI:** 10.1038/s41598-020-65751-1

**Published:** 2020-06-03

**Authors:** Suyun Noh, SungHyun Nam

**Affiliations:** 1 School of Earth and Environmental Sciences, College of Natural Science, Seoul National University, Seoul, 08826 Republic of Korea; 2 Research Institute of Oceanography, College of Natural Science, Seoul National University, Seoul, 08826 Republic of Korea

**Keywords:** Physical oceanography, Physical oceanography

## Abstract

Oceanic internal waves near the local inertial frequency or near-inertial internal waves and internal waves of tidal origin or internal tides are two types of low-frequency internal waves. Their interactions, interaction with background field, and resulting internal waves at higher frequencies beyond the near-inertial and tidal frequencies have rarely been reported despite its importance on ocean mixing and circulation of energy and materials. Here, we present five episodic enhancements of the high-frequency or continuum frequency waves (CFWs) observed in the southwestern East Sea (Japan Sea) and discuss causes for the enhanced CFWs in relation to near-inertial waves (NIWs), semidiurnal internal tides (SDITs), mesoscale flow fields, and their interactions. The NIWs were amplified due to local surface wind forcing, significantly interacting with mesoscale strain via wave capture. The SDITs were generated in a remote place and propagated into the observational site, largely depending on the mesoscale fields. The observational results suggest that the five episodes of CFWs are results of enhanced NIWs or SDITs, or their wave-wave interaction, rather than locally generated lee-waves. Our study suggests the significant impact of mesoscale circulation on the variability of internal waves from near-inertial to buoyancy frequencies through multiple pathways.

## Introduction

Oceanic inertio-gravity waves or internal waves are ubiquitous in the stratified, rotating ocean, and play a key role in providing a significant portion of energy to induce turbulent mixing, and redistributing energy and materials in the ocean^1–6^. Internal waves at time scales from near-inertial to near-buoyancy periods are not always amplified at the same time, nor are their energies spatially homogeneous. They therefore have a potentially important spatio-temporal influence on the distribution and redistribution of energy and materials, and marine ecosystems^7–11^.

Internal waves at a frequency near the local inertial frequency or near-inertial waves (NIWs) are often generated by storm passages, predominantly propagate equatorward^4,12–14^, and are significantly modified through interactions with mesoscale flows^15–21^. The energy exchange between NIWs and mesoscale eddies is believed to be important for the energy budget^5,19,22–27^, but the forcing mechanisms responsible for the process under the wind forcing are not always clear. According to recent studies analysing the energy exchange using a modified slab model (including geostrophic flow) and realistic numerical simulations, a permanent energy transfer from mesoscale eddies to NIWs exists in the presence of strain with a transfer efficiency proportional to the total strain variance during the wind forcing stage^25,26^. Relative vorticity has been suggested to not only induce the permanent energy transfer, but also affect the transfer efficiency in the presence of strain^23,25,26^. Recent studies noted that the strain of mesoscale flow fields plays an important role in NIW and mesoscale energy exchange via the wave capture process, allowing nonlinear interaction between NIWs and the mesoscale field, e.g., drawing NIW energy from the mesoscale flow^17–19,27^. However, such interaction between NIWs and the mesoscale field is not always clear in many seas due to a lack of *in-situ* observations.

Ocean tides generate another type of low-frequency internal wave or internal tide (also referred as baroclinic tides) as barotropic tidal flow (flow associated with surface tides) interacts with bottom topography^28–30^. Diurnal and semidiurnal (SD) internal tides (ITs) are generated when and where their characteristic slope matches the bottom slope, propagate via interaction with background mesoscale conditions, and ultimately dissipate^30–33^. In spite of the tremendous progress on SDITs and diurnal ITs, including global time averaged maps of barotropic to baroclinic conversion and internal tidal beams^34–36^, spatio-temporal variability of local generation, propagation, refraction, and dissipation (or damping) of SDITs and diurnal ITs in many seas, and their interactions are still poorly understood.

Internal waves at higher frequencies (0.09–0.50 cph), defined here as continuum frequency waves (CFWs), have long been described by the classical Garrett-Munk (GM) spectrum^37^ and believed to arise from nonlinear wave-wave interactions transferring energy out of the NIWs and ITs into the broadband continuum^3,38,39^. As the level of the continuum or CFW energy is closely related to small-scale turbulent mixing, many works have been dedicated to better understand the processes underlying the variations of CFW energy and spectral departure from the GM spectrum. Recent studies suggested the relationship between observed mixing rates and internal wave generations along with lee-waves over rough topography, hypothesising that the CFW energy comes only from the wind, tides, and mesoscale turbulence^40–42^. However, our understanding on processes underlying the CFW energy variations in space and time are largely limited due to rare relevant observations.

**Figure 1:**
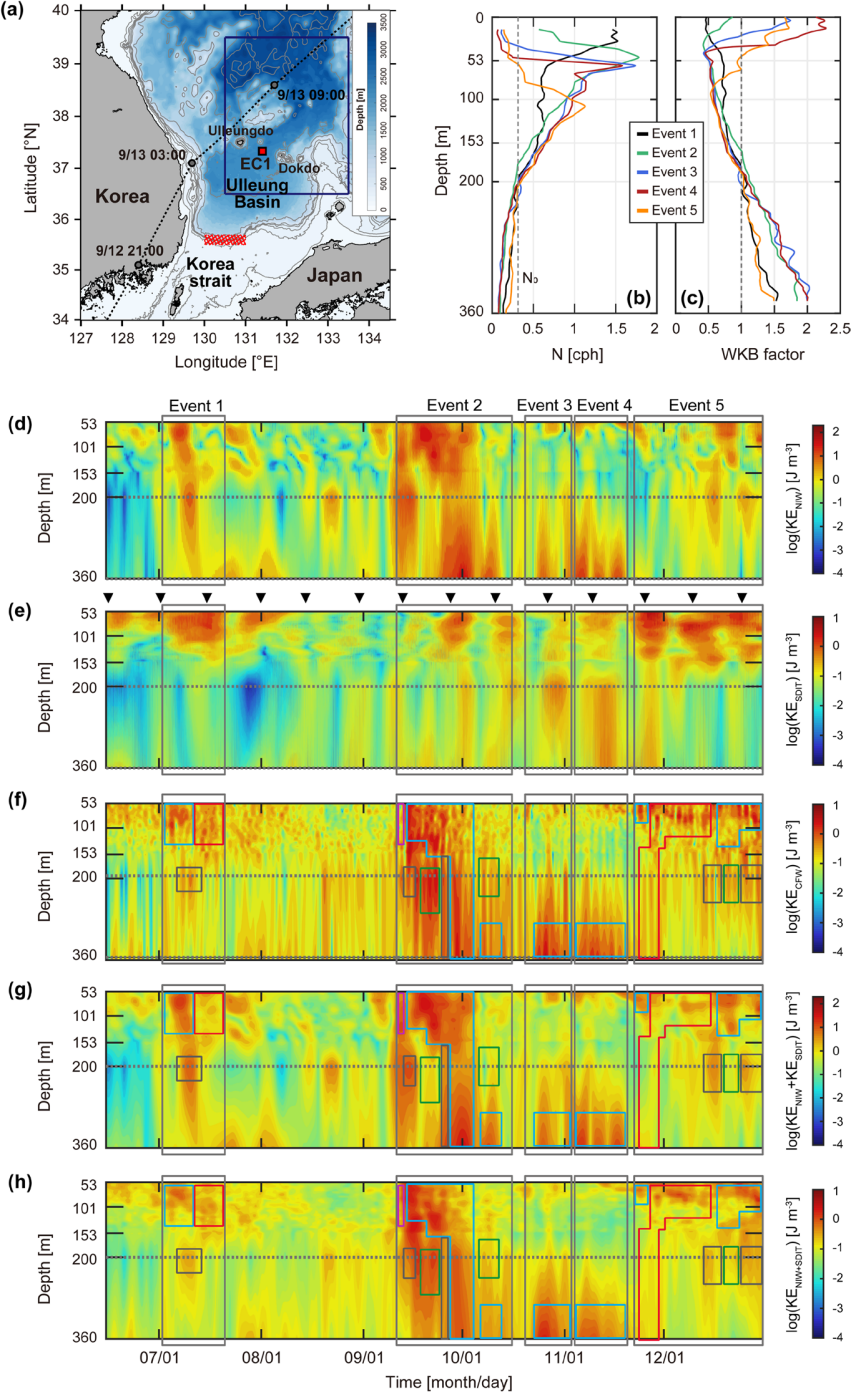
(**a**) Geographic map of the southwestern East Sea (Japan Sea) with bathymetry (colour). Location of a subsurface mooring named EC1 is marked by the red square. Grey circles and dotted line indicate the path of the centre of typhoon Maemi. The red hatched area represents locations where the SDITs can be generated. Wind stress data averaged over the area are denoted with the blue rectangle. Vertical profiles of buoyancy frequency *N* and WKB factor for Events (colours) are shown in (**b**,**c**), respectively. In (**d–h**), time-depth contours of WKB-scaled (**d**) KE_NIW_, (**e**) KE_SDIT_, (**f**) KE_CFW_, (**g**) KE_NIW_ + KE_SDIT_, and (**h**) KE_NIW+SDIT_ are plotted, respectively. The KE_CFW_, KE_NIW_ + KE_SDIT_, and KE_NIW+SDIT_ were 40-hour low-passed. Here, the KE_NIW_ + KE_SDIT_ and KE_NIW+SDIT_ represent summation and interaction (see Table 3) of NIWs and SDITs, respectively, whereas KE_NIW_, KE_SDIT_, and KE_CFW_ are directly estimated from NIWs, SDITs, and CFWs, respectively. Five events are denoted by grey boxes. Each colour box in (**d–h**) represents enhanced NIWs only with no enhancements of SDITs or CFWs (purple), enhanced CFWs only with no enhancements of NIWs or SDITs (green), enhanced NIWs and CFWs with no SDIT enhancement (black), enhanced SDITs and CFWs with no NIW enhancement (red), and enhanced NIWs, SDITs, and CFWs (blue). Timings of spring tide at the nearby tide-gauge station (Busan) are denoted by triangles in (**e**). The figure was generated by S. Noh using MATLAB R2019b, http://www.mathworks.com.

In the southwestern East Sea (also referred as Japan Sea) off the east coast of Korea, episodic events of NIWs and SDITs have been reported^19,31,43–57^. The NIWs generated by local surface wind forcing have widely been observed in the region^49,51,53,54^, yet their interactions with mesoscale field were examined in only few studies^19,44,50,53,56^. A semi-permanent anticyclonic eddy named Ulleung Warm Eddy (UWE) was found to affect the distribution of NIW energy in the region as discussed by *Jeon et al*.^57^. In addition, upward propagating NIWs due to the reflection of downward propagating NIWs back to the surface from the UWE thermostad were observed ^19^. However, the role of mesoscale strain in exchanging energy between NIWs and the mesoscale field has not been investigated thus far. Moreover, the mechanism of NIWs interacting with ITs and enhancing CFWs remained unanswered. Although diurnal ITs are mostly trapped in the southern Ulleung Basin near the generation area, northern slope of the Korea Strait (red hatched area in Fig. 1a), SDITs generated in the same area easily propagate poleward as located in the north of diurnal and south of SD critical latitudes^34,58,59^, interacting with mesoscale circulation^31,45–47^. Thus, this study aims at (1) characterising the time and location of the enhancement of three kinds of internal waves (NIWs, SDITs, and CFWs) in the region, and (2) addressing possible mechanisms to explain the enhanced CFWs in relation to those of NIWs, SDITs, their interactions, and interactions with the mesoscale field.

## Results

### Temporal variations of enhanced internal wave energy over the vertical

Five episodic CFW enhancements (Events 1–5) were observed along with those of NIWs or SDITs between 53 and 360 m at a subsurface mooring named EC1 located in the northern Ulleung Basin from July to December 2003 (Fig. 1a–f, and Table 1). NIW horizontal kinetic energy (KE_NIW_) varies drastically with depth and time after removing stratification effects (Fig. 1b,c) and yields different temporal and vertical structures during the events with the highest KE_NIW_ found during Event 2 (Fig. 1d). In contrast to the NIWs intensified between 53 and 360 m during Events 1, 2, and 5, high KE_NIW_ was rarely observed between 100 and 200 m during Events 3 and 4. Temporal variations of SDIT kinetic energy (KE_SDIT_) basically followed a noticeable fortnightly spring-neap tidal cycle (Fig. 1e). However, vertical KE_SDIT_ structures significantly vary with time, yielding surface intensified features during Events 1 and 5 in contrast to spreading features during Events 2–4 and early Event 5. High KE_SDIT_ was rarely found below 153 m during Event 1 and between 53 and 360 m in August between Events 1 and 2. Not surprisingly, the time-depth pattern of CFW energy (KE_CFW_) was generally similar to those of NIWs, SDITs, their summations (KE_NIW_ + KE_SDIT_), and their interactions (KE_NIW+SDIT_), including the energies at higher tidal harmonics as well as interaction frequencies (Fig. 1d–h).

**Table 1:** Periods of internal wave events identified by 3-day low-passed, WKB-scaled CFW kinetic energy (KE_CFW_) observed at the EC1 in 2003.

Event Number	Period (Month/Day in 2003)
1	July 2–July 21
2	September 11–October 16
3	October 20–November 3
4	November 4–November 20
5	November 22–December 31

High KE_CFW_ in Event 1 was found between 53 and 100 m, and 200 m where high KE_NIW_ or KE_SDIT_ were observed with significantly (*p* < 0.05) high correlation coefficients between KE_CFW_ and KE_NIW_ + KE_SDIT_ and between KE_CFW_ and KE_NIW+SDIT_ (Fig. 1d–g, Table 2). The time-depth pattern of KE_CFW_ during Event 2 was generally more similar to those of KE_NIW_ than KE_SDIT_, but it was complicated by the periods and depths where KE_CFW_ was high without enhanced NIWs or SDITs (green boxes in Fig. 1f–h). Conversely, CFWs at a depth between 53 and 100 m during the early part of Event 2 were not enhanced in spite of high KE_NIW_ (purple box in Fig. 1f–h). During Events 3–4, enhanced CFWs were accompanied by high KE_NIW_, KE_SDIT_, KE_NIW_ + KE_SDIT_, and KE_NIW+SDIT_, with significantly high correlations between KE_CFW_ and KE_NIW_ + KE_SDIT_ except at 153 m for Event 3 and 53 and 360 m for Event 4. Significantly high correlations were also found between KE_CFW_ and KE_NIW+SDIT_ except 77 m for Event 4 (Fig. 1d–g, Table 2). During Event 5, correlations were significant between KE_CFW_ and KE_NIW_ + KE_SDIT_ and between KE_CFW_ and KE_NIW+SDIT_ except 77 m, although the KE_CFW_ at 200 m was high on December 20 without enhanced NIWs or SDITs (green boxes in Fig. 1f–h). Since KE_NIW_ + KE_SDIT_ is highly correlated with KE_CFW_ at all selected depths, there are events and depths where correlations between KE_NIW_ + KE_SDIT_ and KE_NIW+SDIT_ were also significant (Fig. 1g–h, Table 2).

**Table 2:** Correlation coefficients between KE_CFW_ and KE_NIW_ + KE_SDIT_ (top), KE_CFW_ and KE_NIW+SDIT_ (middle), and KE_NIW_ + KE_SDIT_ and KE_NIW+SDIT_ (bottom). Coefficients where p < 0.05 are bolded.

	Depth	Event 1	Event 2	Event 3	Event 4	Event 5	Total
KE_CFW_ vs. KE_NIW_ + KE_SDIT_	53 m	**0.47**	**0.30**	**0.37**	−0.05	**0.25**	**0.31**
	77 m	**0.88**	**0.43**	**0.32**	**0.21**	−0.18	**0.41**
	153 m	0.06	**0.18**	−0.09	**0.73**	**0.22**	**0.42**
	200 m	**0.80**	−0.04	**0.35**	**0.64**	**0.31**	**0.30**
	360 m	−0.38	**0.65**	**0.66**	−0.12	**0.28**	**0.54**
KE_CFW_ vs. KE_NIW+SDIT_	53 m	**0.65**	**0.93**	**0.62**	**0.79**	**0.42**	**0.74**
	77 m	**0.92**	**0.84**	**0.78**	−0.01	**0.69**	**0.83**
	153 m	**0.74**	**0.78**	**0.82**	**0.66**	**0.76**	**0.82**
	200 m	**0.85**	**0.80**	**0.78**	**0.82**	**0.72**	**0.84**
	360 m	**0.79**	**0.78**	**0.85**	**0.70**	**0.83**	**0.87**
KE_NIW_ + KE_SDIT_ vs. KE_NIW+SDIT_	53 m	**0.74**	**0.34**	**0.77**	−0.19	**0.16**	**0.36**
	77 m	**0.93**	**0.35**	**0.52**	**0.52**	−0.19	**0.47**
	153 m	−0.03	**0.09**	−0.27	**0.67**	**0.14**	**0.41**
	200 m	**0.92**	**0.11**	**0.53**	**0.79**	**0.60**	**0.43**
	360 m	−0.41	**0.87**	**0.77**	0.06	**0.50**	**0.57**

### Changes in horizontal kinetic energy spectra

**Figure 2:**
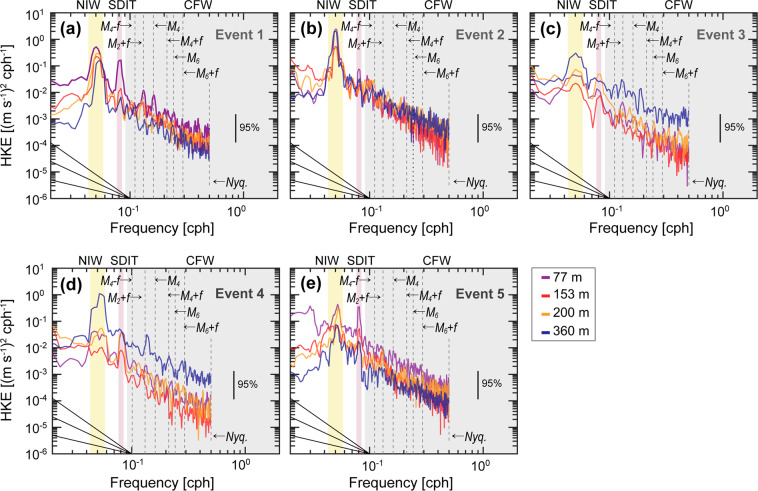
Frequency spectra of horizontal kinetic energy observed at 77 m (purple), 153 m (red), 200 m (orange), and 360 m (blue) during (**a**) Event 1, (**b**) Event 2, (**c**) Event 3, (**d**) Event 4, and (**e**) Event 5, respectively. Diagonal lines in bottom-left corners show fall-off rates or spectral slopes of −1, −2, and −3. Three internal wave bands of NIW (yellow), SDIT (red), and CFW (grey) are shaded with colours. The figure was generated by S. Noh using MATLAB R2019b, http://www.mathworks.com.

The frequency spectra of horizontal kinetic energy at four depths consistently demonstrate temporal variations over the verticals of NIWs, SDITs, and CFWs during the five events (Fig. 2a–e). Spectral peaks at near-inertial (*f*) and SD (*M*_2_) frequencies and their interaction frequencies (e.g., *M*_2_ + *f*, see Table 3) were significant. The spectral energy of the broad near-inertial peak decreased with depth during Event 1, whereas narrower near-inertial peaks had nearly the same spectral energy over depths during Event 2 (Fig. 2a,b). During Events 3 and 4, much broader near-inertial peaks were found with maximum spectral energy at 360 m (Fig. 2c,d). Two spectral peaks at near-inertial and SD frequencies with higher spectral energy at the upper depths found during Event 5 are similar to those during Event 1 (Fig. 2a,e).

**Table 3:** Selected wave-wave interaction frequencies used to reconstruct KE_NIW+SDIT_.

	Frequency	Period (hour)
2* f*	2.00 *f*	9.89
*M*_*2*_ + *f*	2.60 *f*	7.62
*3 f*	3.00 *f*	6.60
*M*_4_	3.19 *f*	6.21
*M*_3_ + *f*	3.39 *f*	5.84
2* f* + *M*_2_	3.60 *f*	5.50
4* f*	4.00 *f*	4.95
*3 f* + *M*_*2*_	4.60 *f*	4.31
*2 f* + *M*_4_	5.19 *f*	3.82
*3 f* + *M*_*4*_	6.19 *f*	3.20

In these spectra, higher energies at the continuum frequency band were found at deeper depths during Events 3 and 4 but at shallower depths during Events 1, 2, and 5, which is consistent with the time-depth patterns of KE_CFW_ (Figs. 1f and 2a–e). The spectral energies integrated over the continuum frequency band were 2.13 × 10^−4^ and 1.14 × 10^−4^ m^2^ s^−2^ cph^−1^ (corresponding to ~0.96 and 0.76 J m^−3^ of KE_CFW_) at 360 m during Events 3 and 4, and 3.74 × 10^−5^, 2.50 × 10^−5^, and 4.94 × 10^−5^ m^2^ s^−2^ cph^−1^ (corresponding to ~0.35, 0.34, and 0.51 J m^−3^ of KE_CFW_) at 77 m during Events 1, 2, and 5, respectively. Spectral slopes at 360 m during the periods of high KE_CFW_ (spectral energy higher than 7 × 10^−5^ m^2^ s^−2^ cph^−1^) were more gentle than the conventional GM spectral slope of −2.00, yielding −1.75, −1.80, −1.86, and −1.40 during late Event 2, Events 3–4, and early Event 5, respectively, while those at 77 m during Events 1, 2, and 5 (−2.27, −2.50, and −2.33) were steeper than the GM spectral slope (Figs. 2 and 4e). During Events 3 and 4, spectral peaks at *M*_2_ + *f* frequency were also significant at 360 m though not significant at 95% confidence interval (Fig. 2c,d).

## Discussion

### NIW generation by local wind forcing

**Figure 3:**
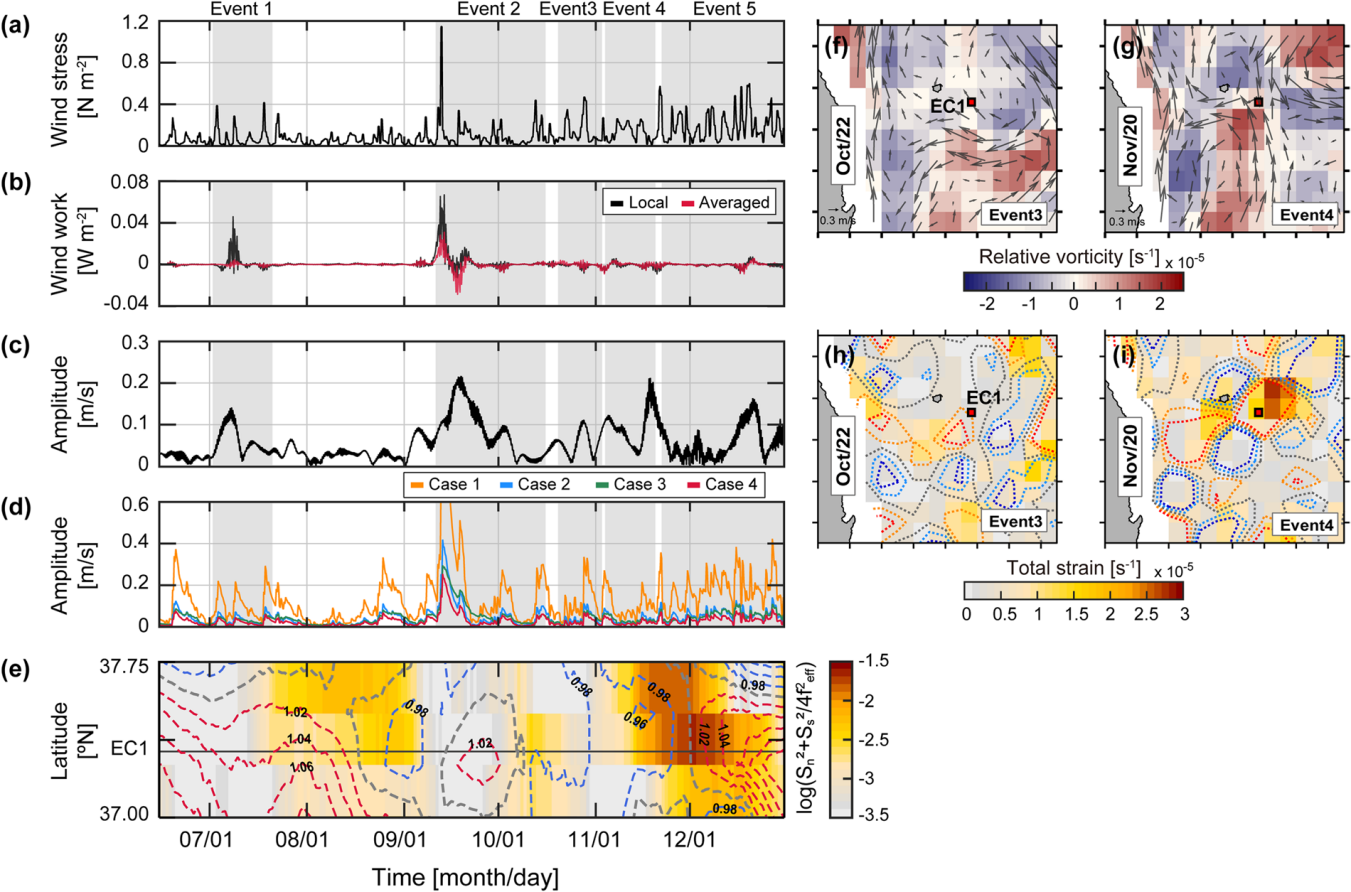
Time series of (**a**) wind stress in N m^−2^ averaged over the area shown in Fig. 1a, (**b**) rate of work done by the surface wind at location nearest to EC1 (black) and averaged over the area (red) in W m^−2^, (**c**) amplitude of NIWs observed at 53 m, (**d**) amplitude of NIWs calculated using the damped slab model for four cases. (**e**) Hovmöller diagram of the energy transfer efficiency in logged colour scale shown in the right at 131.43°E (longitude of EC1) as functions of time and latitude where dashed lines indicate the effective Coriolis frequency normalised by *f* at the surface (contour interval: 0.02 *f*). Maps of (**f**,**g**) surface geostrophic currents derived from satellite altimetry (vectors) and vertical relative vorticity (colours), and (**h,i**) total strain (colours) and Okubo-Weiss parameters *α*^2^ (dotted contours) at −2 × 10^−11^ (blue), −1 × 10^−11^ (cyan), 0.0 (black), 1 × 10^−11^ (orange), and 2 × 10^−11^ (red) for (**f,h**) October 22 and (**g,i**) November 20 corresponding to early Event 3 and late Event 4, respectively. Periods of five events are grey shaded in (**a**–**d**). In (**f**–**i**), the EC1 location is demarcated by the red square. The figure was generated by S. Noh using MATLAB R2019b, http://www.mathworks.com.

Although a simple wind-forced, damped slab model cannot guarantee reproduction of all observed NIWs of surface wind origin, it is useful to identify the episodes of enhanced mixed layer NIWs, e.g., NIWs observed at 53 m (Fig. 3c). Here, the model was not used to reproduce realistic kinetic energy nor its temporal structure but only to identify the events. In particular, it is obvious that the NIWs observed at the upper depths during early Event 2 were triggered by strong wind stress fluctuations (peaked to 1.15 N m^−2^) due to the passage of Typhoon Maemi nearby the observation site (Figs. 1a and 3a). At that time, rate of wind work significantly fluctuated regardless of using local or regional (averaged over the area denoted with the blue rectangle) wind stress (Fig. 3b). The NIWs generated during this particular event were reported by *Nam et al*.^55^, and most (88%) of mixed layer NIWs observed in the region from 1999 to 2004 were suggested to be of wind origin as well reproduced by the wind-forced slab model although the amplitude was systematically over-estimated^54^. Our model applications with four different cases of input parameters along with the rate of wind work confirmed the surface wind-generated NIWs, at least, during Events 1, 2, and 5 (Fig. 3a–d). Note that the vertical direction of NIW energy propagation was downward (or upward phase propagation) based on the time-depth pattern of zonal components of near-inertial currents (not shown) during the Events, consistently indicative of surface energy source.

**Figure 4:**
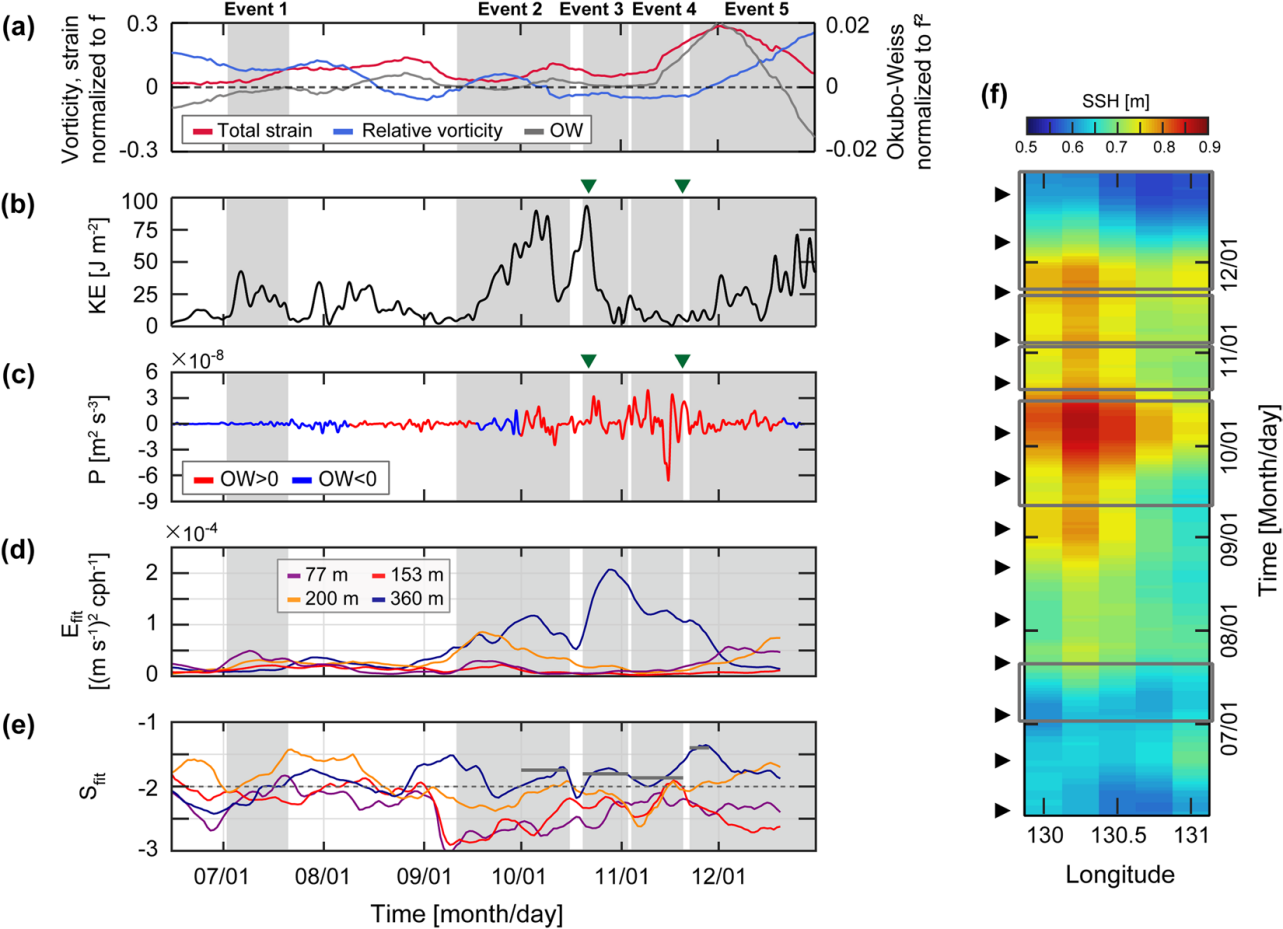
Time series of (**a**) total strain (red, left axis) and vertical relative vorticity (blue, left axis) normalized to *f*, and Okubo-Weiss parameter (thick grey, right axis) normalized to *f* ^2^ at the EC1 location. (**b**) Subinertial kinetic energy averaged from 53 to 360 m. (**c**) rate of energy transfer from mesoscale fields to internal waves. Red (blue) colour denotes positive (negative) Okubo-Weiss parameter *α*^2^. (**d**) Energy level (*E*_*fit*_) and (**e**) slope (*S*_*fit*_) fitted to observed frequency spectrum of horizontal kinetic energy shown in Fig. 2. Horizontal bars in (**e**) indicate the slope averaged over period when *E*_*fit*_ > 7 × 10^−5^ m^2^ s^−2^ cph^−1^ and *S*_*fit*_ > −2. (**f**) Hovmöller diagram (longitude and time plot) of the sea surface height (SSH) at the northern slope of the Korea Strait. Periods of five events are grey shaded in (**a**–**e**). Timings of spring tide at nearby tide-gauge station (Busan, not shown) are denoted by triangles in (**f**). Two selected days for the maps shown in Figs. 3f–i are denoted by green triangles in (**b**,**c**). The figure was generated by S. Noh using MATLAB R2019b, http://www.mathworks.com.

Since the mixed layer NIWs can be amplified by surface background flow field during the generation stage, as recently suggested by *Whitt and Thomas*^25^ and *Jing et al*.^26^, a modified slab model incorporating the effect of background mesoscale flow into the simple model was used to identify the time at which this effect becomes significant. The advection terms in the modified slab model representing nonlinear interaction terms between NIWs and mesoscale flow at the observation site (EC1) were two orders of magnitude lower than the other terms for all events except Event 4. During Event 4, the total strain of surface mesoscale flow consistently increased at EC1 (Fig. 4a) as anticyclonic UWE which existed during Event 3 moved westward and EC1 was located between two cyclonic circulations (Fig. 3f–i). Consistently, the efficiency of energy transfer from mesoscale field to NIWs increased during Event 4 (Fig. 3e), supporting the possibility of mesoscale flow amplifying NIWs in spite of surface wind forcing similar to or weaker than those during Events 1, 2, and 5 (Fig. 3a–d).

### Interaction between mesoscale flow field and NIWs

The KE_NIW_ observed at 360 m of EC1 during Events 3–4 exhibited significantly higher near-inertial spectral energy with a broader peak, implying a source of energy other than the local wind forcing (Figs. 1d, 2c,d, 3d,e, 4a). A Doppler shift by lateral mesoscale flow fields may cause the broadening of the inertial spectral peak^60^. During Events 3–4, mesoscale (subinertial) energy averaged over the depth abruptly decreased from 90 to ~5 J m^−2^, and the total strain of mesoscale flow increased from 0.55 × 10^−5^ to 1.75 × 10^−5^ s^−1^ (Fig. 4a,b). These changes are mainly due to changes of the mesoscale fields (Fig. 3f–i), e.g., the EC1 was located in the western side of the UWE at early Event 3 (October 22) yielding strong geostrophic flow at the location (Fig. 3f) whereas it became located in the middle of mesoscale circulations raising the total strain (Fig. 3g,i). The enhanced total strain supports the possibility of efficiently transferring mesoscale or subinertial energy into NIWs as further evidenced below. Based on the wave capture process suggested previously^16,17,27^, the NIWs undergo the Doppler shift with wavenumber changing exponentially (~ *e*^±*αt*^ where *α*^2^ is an Okubo-Weiss parameter defined as the difference between total strain and relative vorticity of mesoscale flow field), and extract energy from the mesoscale field when and where the strain exceeds vorticity, e.g., *α*^2^ > 0. Events 3 and 4 correspond to the period favouring wave capture at EC1 according to the definition of *Jing et al*.^27^, yielding a positive Okubo-Weiss parameter with positive rates of energy transfer of 3.2 × 10^−9^ and 1.1 × 10^−9^ m^2^ s^−3^, respectively (Fig. 4a,c). Therefore, the NIWs enhanced at 360 m of EC1 during Events 3 and 4 can be explained by the wave capture, indicative of significant energy transfer from the mesoscale field to internal waves.

### SDIT generation at the Korea Strait

Although EC1 is far (~200 km) from the generation area of ITs in the north of the Korea Strait (red hatched area in Fig. 1a), and diurnal ITs (*D*_1_) rarely propagate into the interior of the East Sea as *f* > *D*_1_, SDITs often propagate poleward freely as *f* < *M*_2_. The poleward propagating SDITs can account for high KE_SDIT_ and spectral peaks at *M*_2_, as well as tidal subharmonic frequencies (Figs. 1e and 2a–e). Favourable periods for SDIT generation were found considering the bottom slope and buoyancy frequency at the shelf break in the generation region (corresponding depth of ~200 m)^31^. The internal wave characteristics slope is well matched to the bottom slope in August (between Events 1 and 2) and October (between Events 2 and 3), with buoyancy frequencies of 0.62 and 0.33 cph. On the other hand, the SDITs were weakly generated in June (before Event 1) and December (late Event 5) as the characteristic slope of 0.34–0.90 did not match well to the bottom slope with buoyancy frequencies of 0.04 and 0.20 cph. The summer–fall maximum and spring minimum of the barotropic-to-baroclinic conversion rate of SDITs in the area were consistent with recent numerical results presented by *Jeon et al*.^46^. The generated SDITs reached the EC1 within ~2.5 days, assuming the horizontal speed of mode-1 SDIT (~1 m s^−1^)^31^ in September–November, but not in June, August, and December (as further discussed below). It is reasonable to account for the enhanced KE_SDIT_ and spectral peaks at *M*_2_ and the tidal subharmonic frequencies, particularly below 153 m with poleward propagating SDITs. Although there were general enhancements of KE_SDIT_ following the spring-neap tidal cycle and enhancements of KE_SDIT_ above 153 m, regardless of conditions, for the generation and refraction of SDITs, the KE_SDIT_ below 153 m is affected by the generation of SDITs modulated by the stratification conditions in the northern Korea Strait.

### Interaction between mesoscale field and SDITs

Although SDITs were favourably generated in the northern Korea Strait both in August and October, those in August could not reach the EC1 except in October. The SDITs are refracted westward or eastward and are propagated poleward, or are trapped in the generation area, as the mesoscale fields act as a wave-guide^31,45^. The eastward refraction of poleward propagating SDITs from the generation area into the EC1 is possible only when warmer water with higher sea surface height (SSH) occupies more of the western side than the eastern side of the Korea Strait, yielding faster propagation in the western than the eastern side. This condition was not satisfied before September when the observed SSH in the area was low, indicating that the cold water prevailed in the area to prohibit SDITs from propagating poleward out of the area, i.e., they were trapped in the area (Fig. 4f). During Events 2–4 and early Event 5 (September–November), the SSH in the western side became sufficiently high due to warmer water that allowed the eastward refraction of SDITs towards EC1 (Fig. 4f). Thus, high KE_SDIT_ and spectral peaks at *M*_2_ and tidal subharmonic frequencies below 153 m during Events 2–4 and early Event 5 could be explained by remote SDITs (Figs. 1e, 2b–e and 4e). In particular, SDITs generated at the northern Korea Strait are appropriate to explain the vertically spreading, beam-like KE_SDIT_ well. In contrast, the SDITs generated during August rarely propagate into the EC1 but are trapped within the generation area as relatively cold water (having low SSH) prevails, accounting for the low KE_SDIT_ observed below 153 m of EC1 during Event 1 and between Events 1 and 2 (Figs. 1e and 4f). Nevertheless, the KE_SDIT_ observed above 153 m during same periods was still high, possibly due to long-range propagating low-mode baroclinic (rather than beam-like) SDITs.

### CFW enhancement by interaction between NIWs and SDITs

There are three possible mechanisms for enhanced CFWs: one is forward cascading occurring directly from either one of the enhanced NIWs or SDITs; the second is from nonlinear interaction between NIWs and SDITs; and the third is local generation from the interaction between currents and bottom topography, e.g., Lee-waves. The Froude number, *F*_*r*_ = *Uk*_*H*_/*ω* (*U*: flow speed, *k*_*H*_: horizontally dominant wavenumber of bottom topography, and *ω*: CFW frequency) was considered to examine the third possibility of Lee-wave generation. For a given weak tidal flow and a dominant bottom wavelength of ~7 km (*k*_*H*_ = ~9.0 × 10^−4^ cpm) in the vicinity of EC1 (within a degree), located in a relatively flat area (Fig. 1a), *F*_*r*_ was much smaller than unity. For this reason, we ruled out the third possibility. It is reasonable, considering the correlated time-depth patterns of KE_NIW_, KE_SDIT_, and KE_CFW_, that the wave energy was forward cascaded into the CFWs when and where either NIWs or SDITs or both were enhanced (high KE_NIW_ + KE_SDIT_ and KE_NIW+SDIT_), supporting the first and second possibilities (Fig. 1d–h).

The two mechanisms are not simple, particularly considering the periods and depths where only NIWs were enhanced without enhancements of SDITs or CFWs (purple box in Fig. 1d–h) or where high KE_CFW_ was observed without enhanced NIWs or SDITs (green boxes in Fig. 1f–h). During Event 2, extreme high KE_NIW_ generated by the typhoon passage early on may propagate downward vertically, and presumably equatorward horizontally, as low-mode NIWs with a minimum energy loss into CFWs. A few days later, the CFWs enhanced via remote (rather than local) wave-wave interaction from high-mode NIWs were likely observed in the single mooring without locally enhanced NIWs or SDITs. Note that KE_NIW+SDIT_ at 200 m remained high following high KE_CFW_ during the period when both KE_NIW_ and KE_SDIT_ (thus KE_NIW_ + KE_SDIT_) were low (Fig. 1d–h), implying that wave-wave interaction processes were in action despite there being no local energy source (only remote).

The noticeable time-depth variations of the frequency spectrum of horizontal kinetic energy support different roles of multiple processes in facilitating energy transfer from NIWs and SDITs to CFWs, and potentially to turbulent mixing via forward energy cascading. Time series of energy level *E*_*fit*_ and slope *S*_*fit*_ of the frequency spectra over 20-day-segmented period demonstrates significant deviations from the conventional GM internal wave spectral slope (*S*_*fit*_ = −2.0), as previously recognized^37,61^. *E*_*fit*_ was remarkably high (>7 × 10^−5^ m^2^ s^−2^ cph^−1^) at 360 m during late Event 2, Events 3–4, and early Event 5 presumably due to 1) downward propagating NIWs enhanced by local and regional wind forcing and interaction between NIWs and the mesoscale field, 2) eastward refraction of poleward propagation SDITs into EC1 from the generation area, and 3) enhanced CFWs by interaction between NIWs and SDITs. Significant interactions between NIWs and SDITs and among CFWs are also supported by low *S*_*fit*_ (less than −2.0, indicative of gentler spectral slope that deviates from the GM slope) during the entire observation period, except for Event 1 when KE_SDIT_ was low below 153 m (Figs. 1e and 4d,e).

## Summary and Conclusion

**Figure 5:**
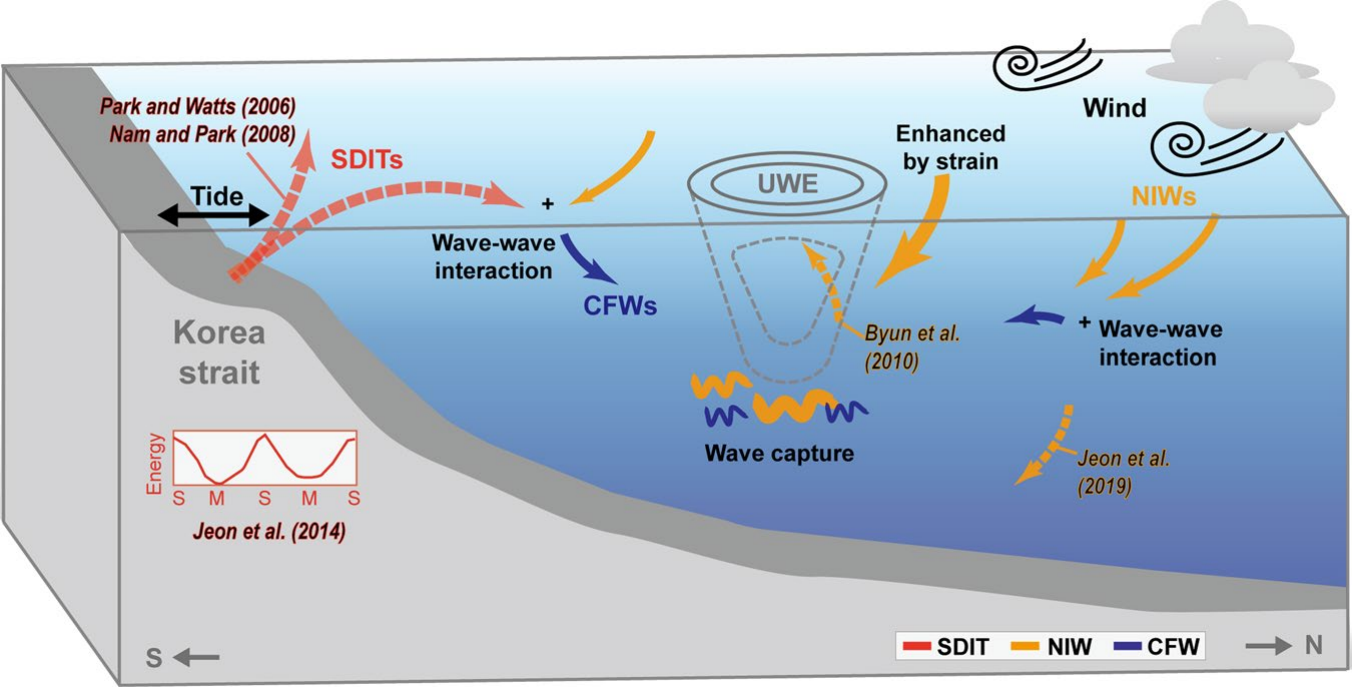
Schematic illustration of mechanisms underlying observed enhancements of internal waves in the southwestern East Sea. The SDITs, NIWs, and CFWs are shown in red, orange, and blue, respectively. The mechanisms relevant to observed enhancements of internal waves discussed in this (solid arrows) and previous (dashed arrows with coloured labels) studies^19,31,45,46,57^. The figure was generated by S. Noh using Adobe illustrator 2019, http://www.adobe.com/.

In summary, we identified five episodic enhancements of CFWs observed from July to December 2003 in the southwestern East Sea, first time in the region, and discussed causes for the enhanced CFWs in relation to NIWs, SDITs, mesoscale fields, and their interactions. The schematics in Fig. 5 depict the impact of the mesoscale circulation on enhanced internal waves from near-inertial to buoyancy frequencies in several different ways. Our findings are summarised as follows:

1. The local wind-forced, damped slab model well reproduced most of mixed layer NIWs supporting well-known mechanisms of surface generation and downward propagation of NIWs, particularly during typhoon passage (Event 2);
2. Modified slab model explained mixed layer NIWs even with weak wind forcing by considering the amplification due to interaction with the surface background flow field during the generation stage (Event 4);
3. Potentially evident and efficient energy transfer from mesoscale field to internal waves via wave capture accounted for enhanced NIWs at 360 m when the total strain exceeds relative vorticity and rates of energy transfer is positive (Events 3 and 4);
4. Remarkable time-depth variations of SDITs in addition to noticeable spring-neap tide cycles were found largely following mesoscale conditions favourable for generating the SDITs at the shelf break in the north of the Korea Strait and eastward refracting of the poleward propagating SDITs toward the observation site in the northern Ulleung Basin (Events 2–4 and early Event 5);
5. Importance of local and remote wave-wave interaction processes and forward energy cascading from NIWs and SDITs into CFWs was emphasised to account for time-depth patterns of KE_CFW_ and the frequency spectrum of horizontal kinetic energy, ruling out the possibility of bottom generation of local CFWs under the condition of small Froude number.

Our observations reveal that CFWs and forward energy cascading from low- to high-frequency internal waves are enhanced not only by direct local and remote wind forcing or by remote tidal forcing, but also through remarkable interactions among the internal waves, and with the mesoscale field via wave capture (Doppler shift of NIWs) and wave guide (refraction of SDITs). The results support previous works, which consistently show that (1) the NIWs are affected by mesoscale strain and vorticity^15–20,25–27,44,57^, (2) ITs are either refracted or trapped by the background fields^31,45–48^, and (3) NIWs and SDITs significantly interact to shape the CFWs via forward energy cascading^4–6,37–39^. More and better observations are required to further deepen our understanding on how the internal waves extract energy from the mesoscale field and transfer the energy into a smaller scale and turbulence in the ocean.

## Methods

### *In-situ* moored measurements of current and hydrography

A subsurface mooring was deployed at an observational site (EC1, 37°19.13′N, 131°25.62′E) located between Ulleungdo and Dokdo in a water depth of 2200 m from November 2002 to April 2004 (Fig. 1a). The mooring was equipped with six single-point rotary-type current meters (RCMs) at 200, 360, 1000, 1365, 1685 and 2235 m, and an upward-looking acoustic Doppler current profiler (ADCP) of four 300 kHz beams at 160 m. Water temperature at 160 m and vertical profiles of horizontal currents at the upper 160 m were measured every hour with a depth interval (bin size) of 4 m (35 bins totally) using the ADCP. The current data collected at shallower than 53 m were not used here because they were presumably contaminated by the diurnal migration of marine biota^62^. Horizontal currents measured every 30 min at the RCMs were subsampled at a 1 h interval. Details on the moored time-series measurements are provided by Kim *et al*.^63^ and *Noh and Nam*^64^. In this study, we used the time-series data collected at the upper 360 m from June 15 to December 31 in 2003.

### Ancillary data

Surface mixed layer depth (MLD) and buoyancy frequency *N* were estimated using Lim *et al*.^65^ that were based on the World Ocean Database 2005 and multi-source hydrographic data. The MLD averaged over the observation period is 34 m having seasonal variation with a typical amplitude of ~20 m. To supplement the EC1 mooring observation, surface geostrophic currents calculated by satellite altimetry-derived SSH of gridded level 4 data were used where horizontal and temporal resolutions are 0.25° and 1 day, respectively. Sea surface wind data derived from spatial blending of QuikSCAT satellite and NCEP reanalysis surface wind data with an interval of 6 h and horizontal resolution of 0.5° were used to estimate wind stress; $\overrightarrow{\tau }=({\tau }_{x},\,{\tau }_{y})$. We used hydrography data collected every second month in 2003 in the generation area of ITs in the north of the Korea Strait.

### Definition of NIW, SDIT, and CFW, and the five events

Three types of internal waves, NIWs $({u}_{NIW},{v}_{NIW})$, SDITs $({u}_{SDIT},{v}_{SDIT})$, and CFWs $({u}_{CFW},{v}_{CFW})$, were defined with cut-off frequencies as [0.85 *f*, 1.15 *f*], [0.95*M*_2_, 1.05*M*_2_] and [1.8 *f*, 8.5 *f*], respectively, where the square brackets [1.8*f*, 8.5*f*] represent lower and upper limits of the waves; *f* and *M*_2_ are local inertial frequency (~0.0505 cph) and SD tidal frequency (~0.0805 cph); *u* and *v* represent zonal and meridional components of horizontal current and subscripts of NIW, SDIT, and CFW denote corresponding waves. To extract three frequency bands, fourth-order Butterworth filters were applied to the hourly time series of the observed horizontal currents (*u, v*) at each depth. To preserve the phase, the filter was applied forward and backward, and the defined bands were not overlapped. The CFW, representing the high-frequency band of internal waves towards the buoyancy frequency, includes interaction frequencies between NIWs and SDITs and among higher tidal harmonics (Table 3).

Horizontal kinetic energies of these waves (KE_NIW_, KE_SDIT_, and KE_CFW_) were computed as $0.5{\rho }_{0}({{u}_{NIW}}^{2}+{{v}_{NIW}}^{2})$, $0.5{\rho }_{0}({{u}_{SDIT}}^{2}+{{v}_{SDIT}}^{2})$, and $0.5{\rho }_{0}({{u}_{CFW}}^{2}+{{v}_{CFW}}^{2})$ in joules per cubic metre (J m^−3^), where *ρ*_0_ is the reference density (=1024.0 kg m^−3^). To minimise the effects of stratification on the kinetic energy, the WKB-scaled ^66^ band-passed currents were used by applying ${u}_{WKB}=u(z,t){\{N(z,t)/{N}_{0}\}}^{-1/2}$, where buoyancy frequency $N={\{-(g/{\rho }_{0})/(d\rho /dz)\}}^{1/2}$, and *z*, *t*, *N*_0_, *g*, and *ρ* are the vertical coordinate, time, reference buoyancy frequency (set to 2.86 cph based on regional observation at the upper 500 m), gravity constant (set to 9.83 m s^−2^) and density, respectively. The WKB factor ${\{N(z,t)/{N}_{0}\}}^{-1/2}$ is less (greater) than unity when and where stratification is stronger (weaker) than the reference (Fig. 1b,c). In this study, five events (Events 1–5) were defined as periods when the 3-day low-passed, WKB-scaled kinetic energy averaged over five depths of 53, 101, 153, 200, and 360 m exceeds 1.0 J m^−3^ (Table 1). The linear sum of the kinetic energies of NIWs and SDITs (KE_NIW_ + KE_SDIT_, Fig. 1g), and their nonlinear interaction (KE_NIW+SDIT_, Fig. 1h), were calculated from KE_NIW_ and KE_SDIT_, as were the kinetic energies at wave-wave interaction frequencies including higher tidal harmonics within CFW range, such as *M*_2_ + *f* (see Table 3). Each frequency component was defined with cut-off frequencies within 5%, e.g., [0.95*(M*_2_ + *f* ), 1.05(*M*_2_ + *f* )], and only components that do not overlap each other were selected to calculate the KE_NIW+SDIT_. The KE_NIW_ + KE_SDIT_, KE_NIW+SDIT_, and KE_CFW_ were all 40-hour low-pass filtered to compute the correlation (Table 2).

### Rate of wind work

To examine wind and current resonance at near-inertial frequency, the rate of wind work was calculated as^57^.

$${\tau }_{{x}_{NIW}}{u}_{NI{W}_{53m}}+{\tau }_{{y}_{NIW}}{v}_{NI{W}_{53m}}$$

where $({\tau }_{{x}_{NIW}},{\tau }_{{y}_{NIW}})$ and (${u}_{NI{W}_{53m}},\,{v}_{NI{W}_{53m}}$) were near-inertial band-passed, zonal and meridional wind stresses at location nearest to the EC1 and averaged over the area shown in Fig. 1a, and zonal and meridional current observed at 53 m, respectively.

### Damped slab model with and without background geostrophic current

To examine the inertial response of the mixed layer to surface wind forcing, a damped slab model was used^67,68^:

$$\frac{\partial {u}_{ML}}{\partial t}=f{v}_{ML}+\frac{{\tau }_{x}}{{\rho }_{0}{H}_{ML}}-r{u}_{ML},$$

$$\frac{\partial {v}_{ML}}{\partial t}=-f{u}_{ML}+\frac{{\tau }_{y}}{{\rho }_{0}{H}_{ML}}-r{v}_{ML}$$

where *H*_*ML*_, *r* and (*u*_*ML*_, *v*_*ML*_) are the MLD, inverse damping time scale, and zonal and meridional currents in the mixed layer, respectively. To test the sensitivity of the slab model results to *H*_*ML*_ and *r*, applications with four different cases were compared (Cases 1–4). The MLD was fixed to 20 and 60 m for Cases 1 and 2 (e.g., *H*_*ML*_ = 20, 60), respectively, whereas time-varying MLD was used for Cases 3 and 4 based on the observations^65^. The damping time scale *r*^−1^ was set to 3 days for Cases 1, 2, and 4, and 6 days for Case 3 based on previous works^49,53,54,57^. Among the modelled results, the modelled amplitudes of NIWs with time-varying *H*_*ML*_ (Cases 3 and 4) were more similar to the observed amplitudes at 53 m than those with constant *H*_*ML*_ (Cases 1 and 2). While the modelled amplitudes of NIWs are sensitive to both MLD and damping time scale, the timing of enhanced NIWs is consistent among the cases, as described in previous section.

Since the mixed layer NIWs can be amplified by the energy transfer from mesoscale flow fields^25,26^, a modified slab model as below incorporating background geostrophic currents, $\overrightarrow{U}=(U,V)$ was used to compare the order of magnitude of the advection terms (second and third terms in the left-hand-side) with other terms:

$$\frac{\partial {u}_{ML}}{\partial t}+{u}_{ML}\frac{\partial U}{\partial x}+{v}_{ML}\frac{\partial U}{\partial y}=f{v}_{ML}+\frac{{\tau }_{x}}{{\rho }_{0}{H}_{ML}}-r{u}_{ML},$$

$$\frac{\partial {v}_{ML}}{\partial t}+{u}_{ML}\frac{\partial V}{\partial x}+{v}_{ML}\frac{\partial V}{\partial y}=-f{u}_{ML}+\frac{{\tau }_{y}}{{\rho }_{0}{H}_{ML}}-r{v}_{ML}$$

### Strain and vorticity of mesoscale field and Okubo-Weiss parameter

Horizontal velocity gradient tensors were calculated from the satellite altimetry-derived surface geostrophic currents $\overrightarrow{U}=(U,V)$ where the normal and shear components of the rate of strain tensor, *S*_*n*_ and *S*_*s*_, and vertical component of the relative vorticity, *ζ* were defined as:

$${\rm{Normal}}\,{\rm{strain}}:{S}_{n}=\frac{\partial U}{\partial x}-\frac{\partial V}{\partial y};$$

$${\rm{Shearstrain}}:{S}_{s}=\frac{\partial V}{\partial x}+\frac{\partial U}{\partial y};$$

$${\rm{Relativevorticity}}:\zeta =\frac{\partial V}{\partial x}-\frac{\partial U}{\partial y}$$

where the subscripts of *U* and *V* represented partial derivatives. Then, the relative importance of total strain and relative vorticity was diagnosed with the Okubo-Weiss parameter^69^, defined as ${\alpha }^{2}=({S}_{n}^{2}+{S}_{s}^{2}-{\zeta }^{2})/4$. The efficiency of energy transfer from mesoscale field to NIWs^26^ is proportional to total strain variance $\sqrt{({S}_{n}^{2}+{S}_{s}^{2})}$ and inverse of the effective Coriolis frequency ${f}_{eff}=\sqrt{{(f+\zeta /2)}^{2}-({S}_{n}^{2}+{S}_{s}^{2})/4}$_._ The rate of energy transfer from the mesoscale field to NIWs was estimated following *Jing et al*.^27^, ${\rm{P}}=-\,0.5(\langle uu\rangle -\langle vv\rangle ){S}_{n}-\langle uv\rangle {S}_{s}$, where the angle brackets 〈〉 represent the running mean over three inertial periods.

### Internal wave characteristic slope

The characteristic slope of internal waves was calculated as^70^:

$$\gamma =\pm \sqrt{({\omega }^{2}-{f}^{2})/({N}^{2}-{\omega }^{2})}$$

where wave frequency *ω* is set to *M*_2_ to compare the characteristic slope of SDITs with the bottom slope at the shelf break in the north of the Korea Strait. Herein, the hydrographic data collected in the northern Korea Strait are used to estimate the time-varying buoyancy frequency.

### Estimation of energy level and slope of frequency spectrum

The frequency spectrum of horizontal kinetic energy was fitted to ${E}_{fit}{\omega }^{{S}_{fit}}$ considering the conventional GM internal wave spectrum, where *E*_*fit*_, *S*_*fit*_, and ω are fitted energy level, slope, and frequencies^29,61^. To estimate temporal variations of the energy level *E*_*fit*_ and slope *S*_*fit*_, a least-square fit of the spectrum for ranging from 0.09 cph and the Nyquist frequency (~0.5 cph) was applied to 20-day-long segment time series of horizontal kinetic energy at given depth. Deviations from the conventional GM internal wave spectrum (*S*_*fit*_ = −2.0) were used to quantify time-depth variations of CFWs.
